# Paradoxes in Borderline Emotional Dysregulation in Adolescence: Influence of Parenting, Stressful Life Events, and Attachment

**DOI:** 10.3389/fpsyt.2021.735615

**Published:** 2021-10-21

**Authors:** Marion Robin, Jean Belbèze, Alexandra Pham-Scottez, Gérard Shadili, Victoire Peres, Jérôme Silva, Maurice Corcos, Mario Speranza

**Affiliations:** ^1^Department of Adolescent and Young Adult Psychiatry, Institut Mutualiste Montsouris, Paris, France; ^2^Medical School, Paris Descartes University, Medical School, Paris, France; ^3^University Hospital Group, Paris Psychiatry and Neurosciences, Paris, France; ^4^Versailles General Hospital, Le Chesnay, France; ^5^Paris-Saclay University, UVSQ, CESP, INSERM, Gif sur Yvette, France

**Keywords:** borderline, adolescent, attachment, alexithymia, parental bonding (PBI), stressful life events

## Abstract

**Introduction:** Borderline personality disorder (BPD) in adolescents is characterized by emotional dysregulation, insecure attachment, a history of stressful life events (SLEs) as well as dysfunctional parent–child interactions. The respective contribution of each of these factors on BPD affective symptoms is not yet clear. The purpose of this study is to assess the distinct impact of parental adversity and SLEs on BPD affective symptoms and the role of attachment and alexithymia in such emotional processes.

**Method:** This study explored parental dysfunction and SLEs as predictors of affective symptoms of BPD and of attachment insecurity in BPD adolescents (*n* = 85) and healthy controls (*n* = 84) aged 13–19 years from the European Research Network on BPD. The links between adversity and BPD symptoms were also investigated by emotional dysregulation assessment, as measured by alexithymia and hopelessness.

**Results:** Dysfunctional parental interactions were linked to affective symptoms, hopelessness, and anxious attachment in healthy controls but not in BPD. Cumulative SLEs were positively correlated with affective symptoms and avoidant attachment in the control group but negatively correlated with both these variables in BPD. Multivariate regression analysis revealed that, in BPD, affective symptoms were independent of dysfunctional parenting but depended on attachment, whereas in controls, a maternal affectionless control style directly predicted affective symptoms. Moreover, increasing numbers of SLEs reduced affective symptoms in BPD, independently of parental interactions or attachment, and were associated with growing use of operative thinking.

**Discussion:** BPD patients showed paradoxical emotional reactions: there was no increase of hopelessness and affective symptoms with an increased parental dysfunction, but a decrease in affective symptoms and hopelessness with cumulative SLE. Two pathways arose, one involving attachment as an emotional dysregulation process for parent–child interactions and a second one for SLE, with a more direct pathway to affective symptoms, independent of attachment but dependent on early interactions, and involving alexithymia. In summary, adversity factors have distinct effects in BPD, and attachment is partly accountable for affective symptoms independently of adversity. Our results suggest that in highly insecure conditions, cumulative adversity may produce paradoxical effects, including a lesser expression of affective symptoms and hopelessness.

## Introduction

Borderline personality disorder (BPD) can be observed in up to 50% of adolescent inpatients (1) and is associated with significant social dysfunction and high levels of suicide and comorbidities, which justifies the importance of preventing this disorder (2). In adults as well as adolescents, emotional dysregulation has been clearly imputed in the onset of the behavioral disorders impacting the severity of borderline disorder, such as suicidal, self-harm, and self-destructive impulsive behaviors (3, 4). Furthermore, the borderline symptoms most directly indicative of this emotional dysregulation, such as anger outbursts and emotional instability (alternating excitement, sadness, and hopelessness), are both the most common symptoms of borderline disorder at this age and those with the greatest predictive value for the illness (1). The description of these symptoms includes paradoxical elements that are very characteristic of BPD adult patients: when faced with minor stress, patients have a disproportionate reaction, while they are sometimes able to manage major stress without any particular reaction (5, 6). Borderline emotional dysregulation clearly involves a discrepancy between the magnitude of the life event that the patient is going through and the internal psychological factors that will give it meaning. The paradox revealed in this particular clinicobiological fact is not really explained to date, and BPD emotional dysregulation is more often described as emotional hyperreactivity than as the combination of hyperreactivity and hyporesponsiveness.

Difficult parent–child interactions as well as other forms of adversity in childhood [abuse, stressful life events (SLEs)] have been associated with the development of BPD in children and adolescents (7, 8). Although the association of adversity and childhood psychopathology is not specific to borderline disorder, a particularly strong effect of maltreatment (abuse and neglect), parental psychopathology (psychiatric disorders, suicide attempts), and dysfunctional parenting have been described in the development of early symptoms of this disorder (9, 10). The entanglement of multiple etiopathogenic levels in a biopsychosocial model of BPD is now well-established, and these social factors play an important role in the development of the disorder and its clinical severity (11, 12). But the relative contribution of these factors (such as SLEs or chaotic family relationships) and psychological dimensions (such as attachment insecurity or alexithymia) on emotional regulation and borderline affective symptomatology is not yet clear (13).

Regarding the psychological dimensions, attachment insecurity has been proposed as a key risk factor for the development of BPD. Bowlby's description of internal operating models (IWMs) applied to self and others emphasizes that individuals who have received inappropriate care from caregivers tend to develop negative IWMs about the self. Conceptually, these IWMs correspond to high levels of attachment anxiety or hyperactivation of the attachment system (14, 15). These individuals fear abandonment, have exaggerated needs for closeness, and are distressed when others are not available (16). In parallel, individuals whose caregivers have not responded to their requests tend to develop negative IWMs of others and to experience high levels of attachment avoidance (or deactivation of the attachment system). As a result, these individuals may develop fears of interpersonal closeness and exhibit compulsive self-sufficiency to avoid rejection (15, 16). Adolescents and adults with borderline disorder tend to oscillate between intense need for care and attempts at self-sufficiency (17) and are characterized by consistent attachment anxiety, which is most closely associated with severe cognitive disorganization and self-dysfunction (18, 19). Along these lines, attachment quantification in BPD patients, by interview as well as self-reported (18, 20), indicates that the attachment types most characteristic of borderline subjects are the fearful and pre-occupied types (21, 22). In each of these attachment types, individuals demonstrate a craving for intimacy and—at the same time—a worry about dependency and rejection. The severity of borderline disorder has also been associated with the “oscillating” form of this disorganized attachment (23).

The inability to identify emotions such as anger and fear in others or in oneself is pointed out as one of the possible explanations for borderline emotional dysregulation (11). Difficulties in identifying one's own emotions refer to the concept of alexithymia. This describes a personality modality characterized by a failure to identify emotions and a difficulty in describing feelings, a lack of imagination, and a tendency to develop concrete and externally oriented thinking (24, 25). The association between borderline symptomatology and alexithymia is fairly well-established by several studies that describe a link between the two dimensions, although it is not restricted to BPD (26, 27). Alexithymia represents a transdiagnosis overlap, also described, for example, in anorexia nervosa, in autism spectrum, or at the interface between the autism spectrum and BPD functioning (28, 29).

The proximal/distal model proposed by Fonagy suggests that distal causes such as trauma or mirroring failure in early life are combined with more proximal causes such as stress sensitivity and attachment arousal. However, it is not yet known how these factors interact or what their independent or cumulative effects are on emotional regulation and clinical symptoms of borderline disorder (13). As attachment is at the crossroads of the gene–environment interaction, it seems interesting to distinguish and explore the respective implications of adversity and attachment styles in the affective symptomatology of BPD.

To this end, we sought to explore the respective influence of adversity—parental dysfunction and SLEs—on affective symptoms, hopelessness, and alexithymia, with the hypothesis that the alteration of the attachment system in BPD explains the dysregulation of emotions, independently of the environmental context. The second hypothesis is that borderline patients have developed a phenomenon of habituation to stress, disconnecting them from their emotions.

## Methods

The study sample included 85 BPD adolescents and 84 healthy controls (HCs) and was recruited within the European Research Network on BPD whose aim was to explore BPD psychopathology in adolescence (30). All subjects completed a research protocol, consisting of a diagnostic evaluation of Axis I, Axis II disorders, and BPD affective symptoms and a self-administered questionnaire to collect sociodemographic and psychopathological data, including parental bonding, SLEs, attachment, and emotions.

Borderline patients (*n* = 85, 87% girls) were recruited in five adolescent-specialized academic psychiatry departments in Belgium, Switzerland, and France. They were considered for inclusion if they presented BPD according to their psychiatrist and were included if they had five or more BPD symptoms according to the Structured Interview for the *Diagnostic and Statistical Manual of Mental Disorders* (*DSM*) personality disorders (SIPD-IV), which has shown good psychometric properties in adolescents (31). Five clinicians, experienced in the assessment of *DSM-IV* Axis I and II disorders in adolescents, conducted the diagnostic interviews (interrater reliability was good, *K*_BPD_ = 0.84). After expressed parental consent, patients received the self-administered questionnaire by mail or directly if they were hospitalized. They returned the completed questionnaire on the day they were interviewed by the investigator. Parents were not interviewed and were not present during the interview. In accordance with the legal status of the study, only participants in the control group received compensation for their participation.

Psychiatric comorbidity was explored using SIPD-IV for Axis II disorders. For Axis I disorders, a semistructured interview assessing *DSM-IV* criteria was used (Kiddie—Schedule for Affective Disorders and Schizophrenia, Kiddie-SADS). Adolescents with a diagnosis of schizophrenia or with any chronic and (or) life-threatening medical illness and adolescents with a mental retardation were excluded from the sample. The patient group included 67.1% of inpatients and 32.9% of outpatients, and 95.6% patients were currently under psychotropic medication. All subjects from the BPD group had at least one Axis I disorder. Mood disorders and anxiety were the most frequently observed comorbidities (63.6 and 63.5%, respectively) followed by eating disorders (36.3%), post-traumatic stress disorder (19.8%), disruptive behavior disorders (9.1%), and substance use disorders (9.1%). The most frequent personality disorder diagnosed in the BPD group was obsessive–compulsive (32%), followed by avoidant (14%), dependent (9%), antisocial (9%), and paranoid (4.5%).

In order to compare BPD patients with normal adolescents, participants in the HC group (*n* = 84) were recruited *via* advertisements placed in schools and universities of comparable geographical areas and were individually matched for gender and age. The procedure with this sample was identical to that of the clinical sample, and HCs were excluded if they were positive for a BP or if they had a diagnosis of current or lifetime mental disorder (according to the Kiddie-SADS), mental retardation, or any chronic or life-threatening medical illness. Axis I and Axis II measurements in the control group revealed the following rates of morbidity: 13.6% mood disorders, 13.6% of oppositional defiant disorders, 9% of anxiety, and 2.3% of obsessive–compulsive personalities. None of the control participants reported any current psychotropic medication use.

Patients and HCs were matched for gender and age (87% of girls in BPD group, mean age_BPD_ = 16.5 years old). A comparison of socioeconomic status using a dimensional index (from higher managerial/administrative professions, to intermediate professions, and unemployed and retired) revealed no difference between the two groups [M_HC_ = 2.48, standard deviation (SD) = 0.51; M_BPD_ = 2.30, SD = 0.71; *p* = 0.29]. Fifty-five percent of the parents of BPD adolescents were divorced vs. 24% of HCs; 28% of BPD patients were out of school (vs. 3% of controls).

The reference ethics committee approved the study (Authorization No. 0611259, Hôtel-Dieu Paris). Freely given, informed consent to participate in the study was obtained from all participants and their parents.

### Measures

The Parental Bonding Instrument (PBI) is a self-administered questionnaire, which is widely used to measure the subjective experience of parent–child bonding from the child's point of view (32). It is a 4-point Likert scale, including 25 items on the maternal bond and 25 items on the paternal bond, with 13 “control” and 12 “care” items for each (Cronbach α coefficient: 0.98). These items are used to generate Care and Control scores for each parent (ranging from 0 to 39 for Control and 0 to 36 for Care). In addition to generating Care and Control scores for each parent, parents can be effectively “assigned” to one of four categories: “optimal parenting” (high care and low control), “affectionless control” (ALC) (low care and high control), “affectionate constraint” (high care and high control), and “neglectful parenting” (low care and low control).

SLEs were evaluated with a questionnaire including 20 experiences: being separated from a parent for at least a month before the age of 1 year, being separated from both parents for at least a year, a history of parental chronic disease (physical or psychological), a history of parental suicide attempt, death of the mother, death of the father, death of a close relative, parental separation or divorce, adoption, interruption of schooling, termination of schooling, relocation, interruption of vocational training, end of vocational training, judicial interpellation, placement in a home or in foster care, breakup of a love relationship, abortion, accident, and disaster. These experiences (quoted yes/no) were added in a sum score from 0 to 20 (Cronbach α coefficient: 0.97).

Attachment was measured with the Relationship Scales Questionnaire, which is a 30-item scale scored on a 5-point Likert scale, measuring the two main dimensions of attachment: anxiety and avoidance (33). Convergent validity and discriminant validity, as well as construct validity of anxious and avoidant dimensions, have been demonstrated (34). Its measured value of Cronbach α coefficient was 0.97.

The 20-item Toronto Alexithymia Scale (TAS-20) (35, 36) is a 5-point Likert-scale questionnaire (Cronbach α coefficient: 0.98). The 20 items of the TAS are clustered into three factors corresponding to the theoretical dimensions of alexithymia: difficulty identifying feelings (DIF), difficulty describing feelings (DDF), and externally oriented thinking (EOT). TAS-20 scores are reliable, and the three-factor structure is replicable (25, 35). The TAS-20 is currently the most widely used measure of alexithymia, and considerable work has gone into testing its reliability and validity (35–37).

The BHS-Beck Hopelessness Scale is a 20-item true/false self-report, which measures the intensity of the subject's hopelessness (38). Its measured Cronbach α coefficient was 0.97.

BPD affective symptoms were evaluated with the Diagnostic Instrument for Borderline—Revised (DIBR) (39, 40). This is a semistructured interview, which evaluates quantitatively and qualitatively the main clinical dimensions of BPD: affects, cognition, impulsive actions, and interpersonal relationships (Cronbach α coefficient: 0.99). Each domain includes items rated at three levels: 0 (negative response), 1 (probable response), or 2 (positive response). The different items are grouped into summary statements (5 for affects and impulsive actions, 3 for cognition, and 9 for interpersonal relationships). The summary statements are then transformed into scaled scores according to a specific algorithm and added together to obtain a final total DIBR score, from 0 to 10. In the present study, we extracted the DIBR-affect subscale, in order to explore the affective axis of BPD.

### Statistical Analysis

All variables, including adversity experiences (SLEs and PBI), affective symptoms (DIBR-affect), attachment (RSQ), and emotional regulation (BHS, TAS), were compared between BPD and HC groups. Mean scores of these scales were compared using *t*-tests after verification of normal distribution of the samples. Bivariate correlations were thus conducted between adversity experiences and all other variables. ALC, the most pathogenic parenting style, characterized by low care and high control, both of which are frequently present in borderline pathology (32), was also included in the analyses in order to investigate its effect and to simplify analyses.

To assess the respective association of adversity and attachment on affective symptoms, multivariate regression models predicting affective symptoms were performed in BPD and HC groups separately. As the contribution of attachment in psychopathology is still to be investigated, two types of models were designed, in order to distinguish adversity effects on affective symptoms and on attachment. Models 1, 2, 3, and 4 predicted affective symptoms, whereas models 5 and 6 predicted attachment dimensions. Model 1 tested the respective effect of adversity experiences—SLEs and PBI-ALC from mother and father—on affective symptoms. Model 2 tested the respective effects of maternal PBI-ALC and anxious and avoidant dimensions of attachment on affective symptoms. Model 3 tested the respective effects of paternal PBI-ALC and anxious and avoidant dimensions of attachment on affective symptoms. Model 4 tested the respective effects of SLEs and anxious and avoidant attachment on affective symptoms.

Model 5 tested the respective effects of maternal PBI-ALC, paternal PBI-ALC, and SLEs on the anxious dimension of attachment. Model 6 tested the respective effects of maternal PBI-ALC, paternal PBI-ALC, and SLEs on the avoidant dimension of attachment. Because of missing data for some BPD patients, multivariate analyses were performed on 47 to 56 patients, depending on the variables included in each model. Because of the sample size and missing data, all models include a maximum of three variables. All variables included in the models were standardized.

## Results

### Comparisons Between BPD and HCs

As expected, adversity events occurred more frequently in BPD patients, compared to HCs. Similarly, BPD patients had significantly higher scores of both attachment anxiety and attachment avoidance than matched controls. Hopelessness, DIBR, and alexithymia were also more intense in BPD than in HCs. Results are reported in Table 1.

**Table 1:** Comparison between BPD and HC groups on parental bonding, stressful life events, attachment, affective symptoms, hopelessness, and alexithymia.

	**BPD** **(***n*** = 85)**		**HC** **(***n*** = 84)**		
	**Mean**	**(sd)**	**Mean**	**(sd)**	**Cohen's d**
Age	16.5	(1.4)	16.1	(1.5)	0.26
SLE	4.1	(2.1)	1.6	(1.1)	1.56^***^
Maternal care	23.2	(8.7)	28.4	(6.6)	−0.689^***^
Paternal care	16.7	(9.9)	25.6	(7.4)	−1.05^***^
Maternal control	16.0	(8.5)	12.2	(6.7)	0.505^**^
Paternal control	14.9	(7.3)	9.6	(5.4)	0.842^***^
Anxiety score	−7.4	(6.4)	−1.6	(6.4)	0.908^***^
Avoidance score	−6.7	(9.8)	−2.6	(6.7)	0.497^**^
DIBR	8.6	(2.0)	2.4	(2.4)	2.87^***^
BHS	10,0	(5.2)	5.1	(3.6)	1.14^***^
TAS total	59.6	(10.4)	49.1	(9.8)	1.05^***^
DIF	22.7	(5.9)	15.5	(5.4)	1.28^***^
DDF	17.4	(4.4)	14.1	(4.2)	0.75^***^
EOT	19.5	(4.8)	19.4	(4.3)	0.02

*BPD, borderline personality disorder; HC, healthy controls; SLE, stressful life events; DIBR, Diagnostic Interview for Borderline—Revised; BHS, Beck Hopelessness Scale; TAS, Toronto Alexithymia Scale; DIF, difficulty identifying feelings; DDF, difficulty describing feelings; EOT, externally oriented thinking*.

*t-test significance*:

** *p < 0.01*;

*** *p < 0.001*.

### Correlations Between Adversity, Affective Features, and Attachment in BPD and HCs

#### Parental Bonding

**Figure 1:**
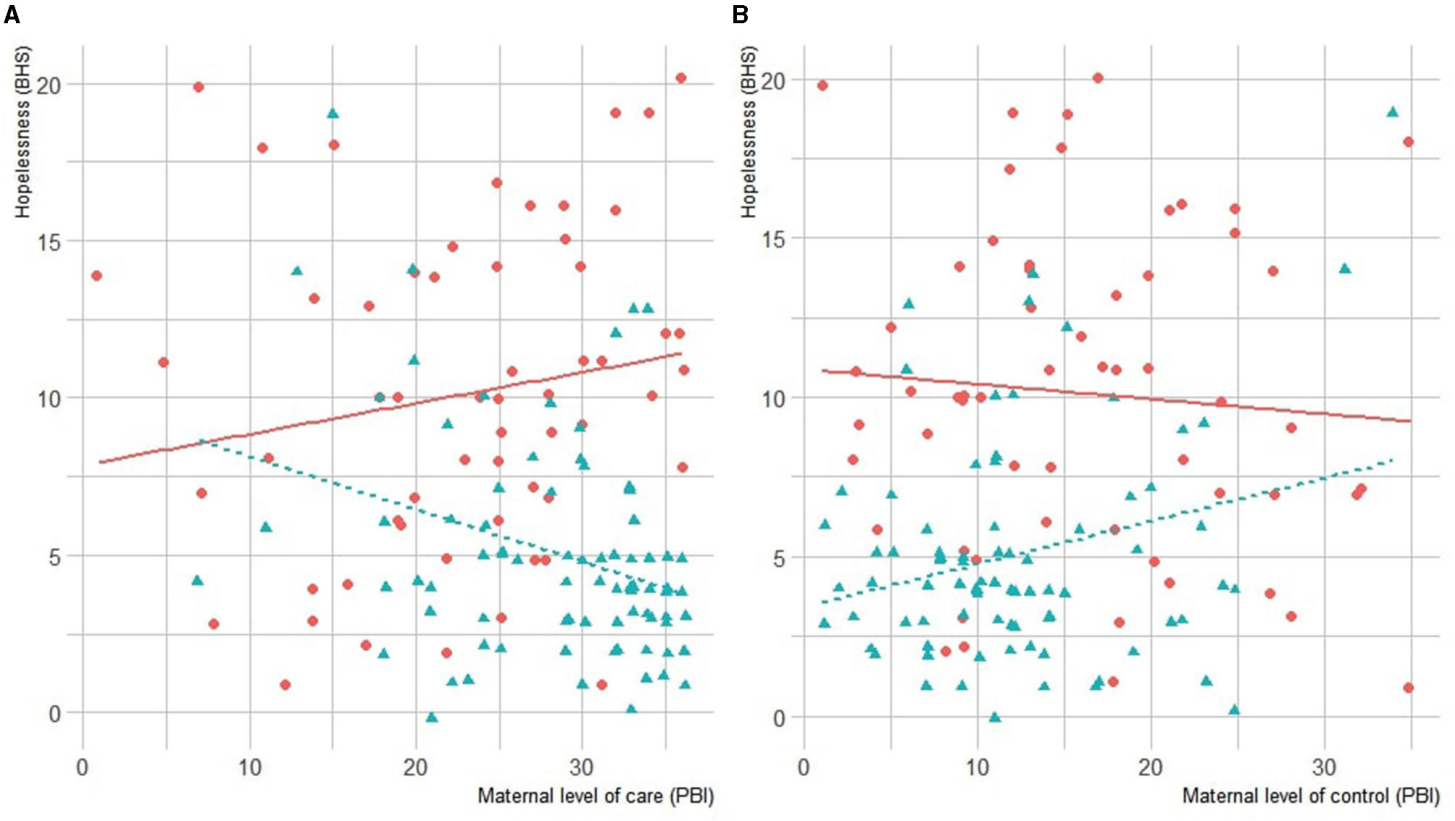
**(A)** Correlation between maternal care and hopelessness. **(B)** Correlation between maternal control and hopelessness in BPD and HC groups. BPD scores are represented in blue, and HC's in red. BHS, Beck Hopelessness Scale; BPD, Borderline Personality Disorder; HC, healthy controls.

As shown in Table 2, dysfunctional maternal bonding (care and control) was associated with affective symptoms and hopelessness in HCs but not in BPD patients. These results are reported in Figure 1A. A decreased paternal care was also correlated with affective symptoms in HCs but not in BPD patients.

**Table 2:** Correlations between adversity, affective symptoms, hopelessness, and attachment in BPD and HC groups.

***n*** **= 80**	**DIBR-affect**	**BHS**	**Anxious score**	**Avoidant score**
**HC GROUP**				
PBI maternal care	−0.43^***^	−0.31^**^	−0.2	0.084
PBI paternal care	−0.3^**^	−0.18	−0.27^*^	−0.2
PBI maternal control	0.24^*^	0.25^*^	0.36^***^	−0.13
PBI paternal control	0.0053	0.14	0.14	0.1
PBI maternal ALC	0.27^**^	0.059	0.25^**^	−0.043
PBI paternal ALC	0.083	0.068	0.054	0.027
SLE	0.25^*^	0.13	−0.093	0.22^*^
***n*** **= 59**	**DIBR-affect**	**BHS**	**Anxious score**	**Avoidant score**
**BPD GROUP**				
PBI maternal care	0.12	0.17	−0.13	0.025
PBI paternal care	0.2	−0.17	0.16	−0.13
PBI maternal control	−0.025	−0.078	0.00	0.00
PBI paternal control	−0.16	0.12	0.05	−0.034
PBI maternal ALC	−0.062	−0.089	−0.075	0.17
PBI paternal ALC	−0.1	0.24	0.073	−0.06
SLE	−0.43^***^	−0.38^**^	0.015	−0.33^**^

*BPD, borderline personality disorder; HC, healthy controls; DIBR, Diagnostic Interview for Borderline—Revised; BHS, Beck Hopelessness Scale; PBI, Parental Bonding Instrument; SLE, stressful life events*.

* *p < 0.05*;

** *p < 0.01*;

*** *p < 0.001*.

#### Stressful Life Events

**Figure 2:**
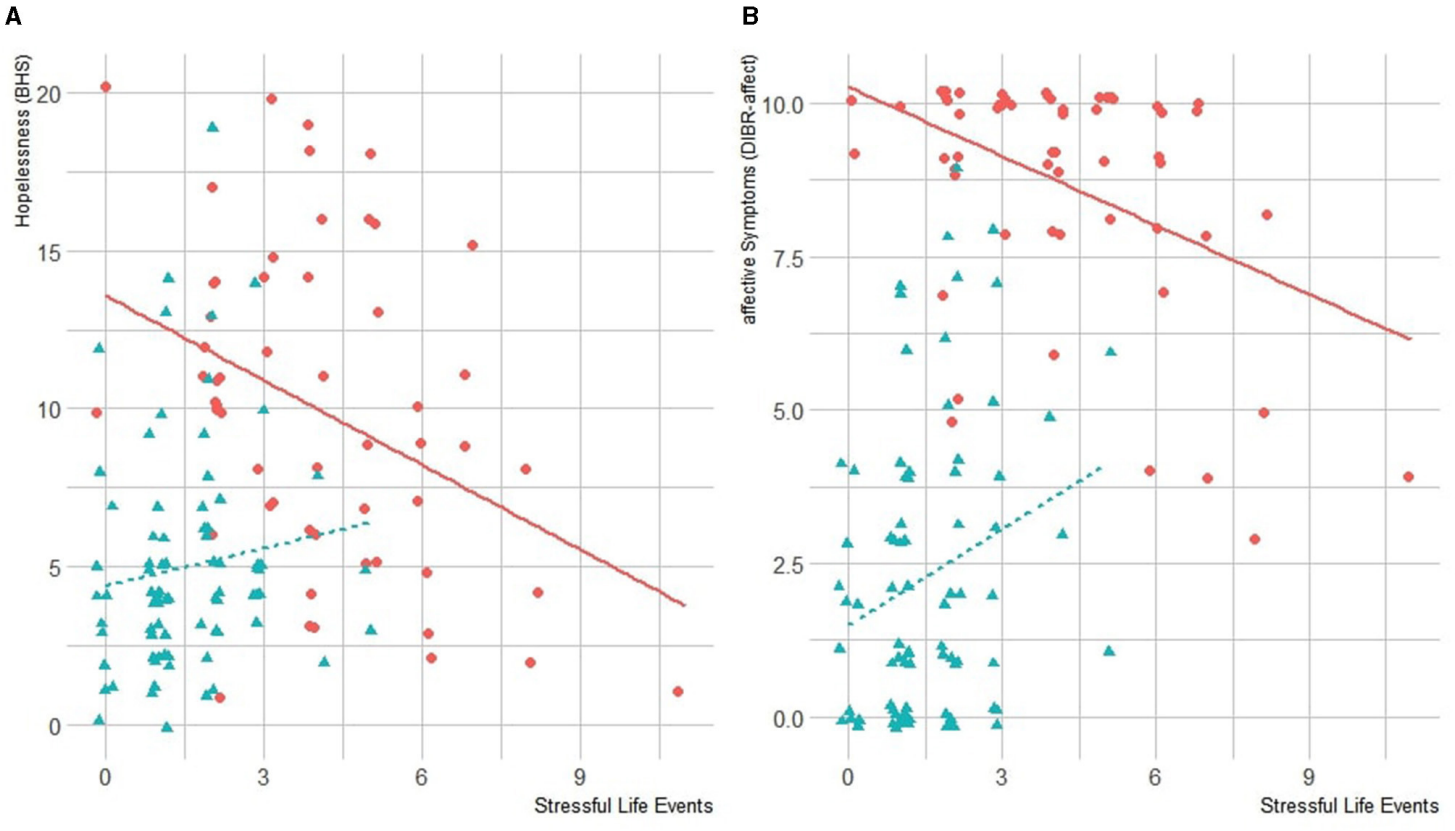
**(A)** Correlation between SLEs and hopelessness. **(B)** Correlation between SLE and DIBR-affect in BPD and HC groups. BHS, Beck Hopelessness Scale; BPD, borderline personality disorder; DIBR, Diagnostic Interview for Borderline—Revised; HC, healthy controls; SLE, stressful life events.

The SLE score was significantly correlated with affective symptoms in HCs, but not with hopelessness. It was, however, associated with a reduction of hopelessness and affective symptoms in the BPD group. These results are reported in Figure 2.

#### Attachment

In the HC group, a reduction in paternal care was associated with anxious attachment. A maternal controlling behavior was associated with anxious attachment, and SLEs were associated with avoidant attachment. In BPD, attachment was not associated with parental care, and SLEs were negatively associated with avoidant attachment.

In summary, affective symptoms were associated with both parental interactions and SLEs in HCs. In BPD, only SLEs were associated with affective symptoms, and the association was negative. Avoidant attachment was associated with SLEs in both HCs and BPD but with an opposite effect. Anxious attachment was associated with parental care in HCs only.

#### Toronto Alexithymia Scale

**Figure 3:**
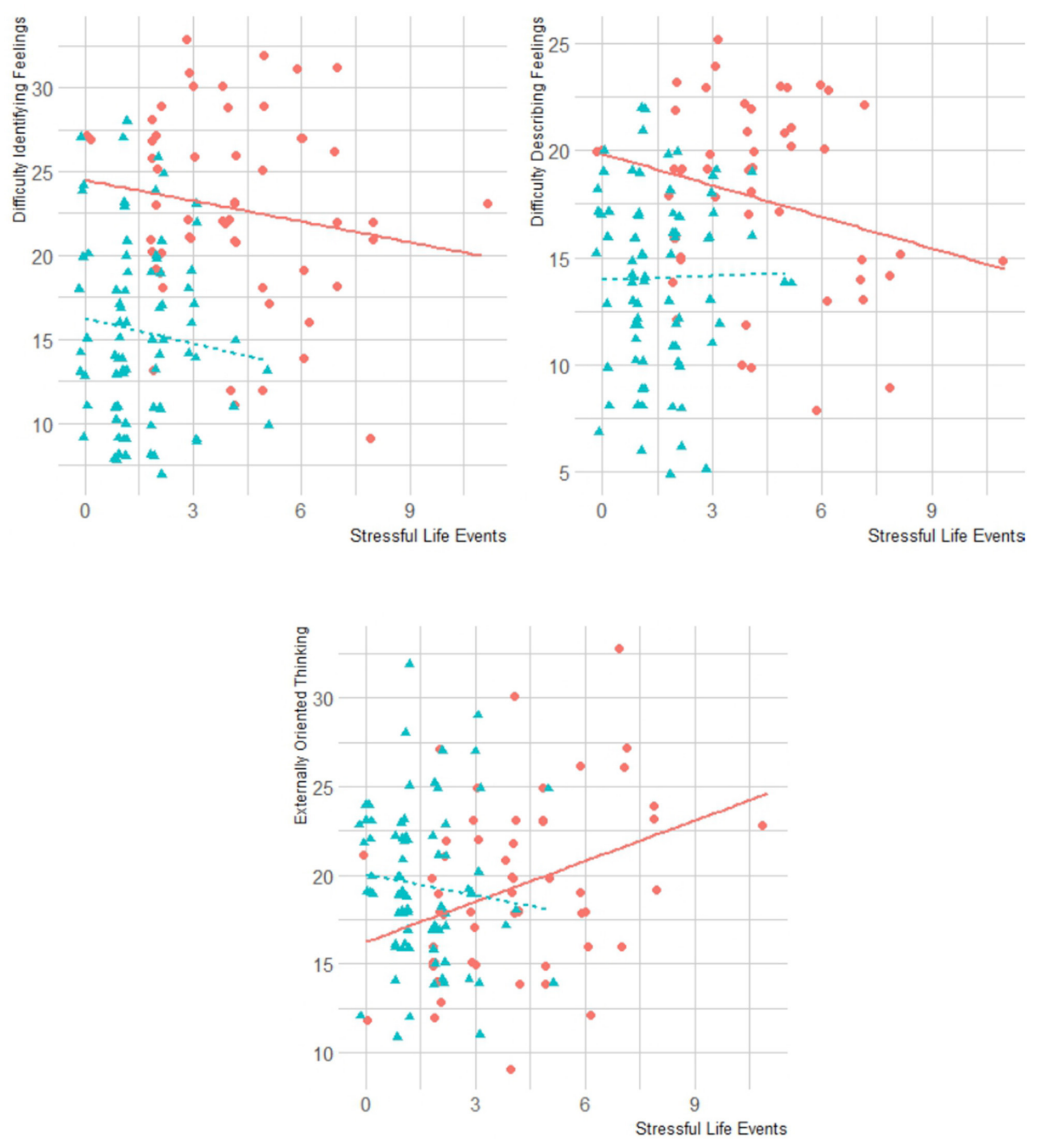
Correlations between alexithymia (total, DIF, DDF, EOT) and SLEs in BPD and HC groups. BPD, borderline personality disorder; HC, healthy controls; TAS, Toronto Alexithymia Scale; DIF, difficulty identifying feelings; DDF, difficulty describing feelings; EOT, externally oriented thinking.

As shown in Table 3, a reduction in both paternal and maternal care in HCs was associated with a difficulty in identifying feelings (TAS-DIF). A difficulty in describing feelings (TAS-DDF) was negatively associated with paternal care in BPD. SLEs were negatively associated with DDF and positively associated with the external oriented thought of the TAS (TAS-EOT) in BPD. These results are reported in Figure 3.

**Table 3:** Correlations between adversity and alexithymia in BPD and HC groups.

***n*** **= 80**	**TAS total**	**TAS-DIF**	**TAS-DDF**	**TAS-EOT**
**HC GROUP**				
PBI maternal care	−0.31^**^	−0.28^**^	−0.18	−0.16
PBI paternal care	−0.24^*^	−0.22^*^	−0.16	−0.12
PBI maternal control	0.21^*^	0.18	0.16	0.1
PBI paternal control	0.15	0.058	0.12	0.16
Stressful life events	−0.096	−0.1	0.016	−0.11
***n*** **= 59**	**TAS total**	**TAS-DIF**	**TAS-DDF**	**TAS-EOT**
**BPD GROUP**				
PBI maternal care	0.12	0.014	0.13	0.11
PBI paternal care	−0.28^*^	−0.16	−0.25^*^	−0.16
PBI maternal control	0.08	0.043	−0.088	0.2
PBI paternal control	−0.098	−0.022	−0.11	−0.08
Stressful life events	−0.03	−0.16	−0.26^*^	0.34^**^

*BPD, borderline personality disorder; HC, healthy controls; SLE, Stressful Life Events; TAS, Toronto Alexithymia Scale; DIF, difficulty identifying feelings; DDF, difficulty describing feelings; EOT, externally oriented thinking*.

* *p < 0.05*;

** *p < 0.01*.

#### ALC Model of Parenting Style

A maternal ALC was associated with affective symptoms and anxious attachment in HCs. No association was found for paternal ALC with any of the emotional features and attachment. In BPD, no association was found for maternal or paternal ALC with attachment or emotional manifestations. These results are reported in Table 2.

### Models

#### Effects of Adversity on Affective Symptoms and on Attachment

As reported in Table 4, all models revealed differences between BPD and HCs, except model 3. In model 1, maternal ALC was associated with affective symptoms (DIB-R affects) in HCs. In the BPD group, SLEs were associated with DIB-R affects. When analyzing maternal ALC and attachment dimensions on DIB-R affects (model 2), and maternal ALC was associated with DIB-R affects only in HCs, whereas anxious attachment was associated with DIB-R affects in both BPD and HCs. Avoidant attachment was associated with DIB-R affects in BPD only. Regression analysis of paternal ALC and attachment on affective symptoms revealed no differences between BPD and HCs (model 3), but revealed a specific role of anxious attachment on affective symptoms. SLEs were positively associated with affective symptoms regardless of attachment in HCs. In BPD, a negative association was found, next to the proper role of anxious attachment. Finally, the regression analysis of adversity on attachment (models 5 and 6) revealed that maternal ALC was positively associated with the avoidant dimension of attachment in the BPD group only, whereas paternal ALC was positively associated with the anxious dimension of attachment; SLE scores did not reveal any independent effect on attachment in this model (Table 2).

**Table 4:** Multivariate regression model predicting the respective effects of maternal and paternal ALC and SLEs on affective symptoms and on attachment, in BPD and HC groups.

**HC group**	**DIBR_Affect**	**DIBR_Affect**	**DIBR_Affect**	**DIBR_Affect**	**Anxious score**	**Avoidant score**
	**(model 1)**	**(model 2)**	**(model 3)**	**(model 4)**	**(model 5)**	**(model 6)**
(Intercept)	1.33	0.43	0.16	0.95	−1.67	−0.43
Mother ALC	0.75 ^*^	0.64 ^*^			0.48	−0.41
Father ALC	0.16		0.24		0.04	0.40
SLE	0.76			1.05^*^	−0.06	0.34
Anxious score		0.75^*^	0.88^**^	0.90^**^		
Avoidant score		0.44	0.42	0.34		
Observations	78	80	80	79	78	78
R^2^/R^2^ adjusted	0.173/0.116	0.229/0.176	0.188/0.133	0.215/0.162	0.047/−0.019	0.110/0.048
AIC	354.972	358.010	362.127	355.529	219.708	193.713
**BPD group**	**DIBR_Affect**	**DIBR_Affect**	**DIBR_Affect**	**DIBR_Affect**	**Anx Attacht**	**AvoidAttacht**
(Intercept)	6.98^*^	6.37	7.99^*^	7.29^*^	1.42	−3.82
Mother ALC	0.08	−0.12			−0.19	0.86^*^
Father ALC	−0.30		−0.16		0.68^*^	−0.61
SLE	−0.79^**^			−0.73^**^	0.09	−0.13
Anxious score		0.80^**^	0.62^*^	0.78^**^		
Avoidant score		0.50^*^	0.30	0.25		
Observations	50	56	51	55	47	47
R^2^/R^2^ adjusted	0.171/0.076	0.228/0.151	0.127/0.030	0.340/0.273	0.198/0.100	0.173/0.072
AIC	203.233	230.874	209.045	218.984	130.980	150.662

*BPD, borderline personality disorders; HC, healthy controls; ALC, affectionless control; DIBR, Diagnostic Interview for Borderline—Revised; SLE, stressful life events; AIC, Akaike Information Criteria*.

*Controlled by age and sex*.

* *p < 0.05*;

** *p < 0.01*.

## Discussion

The aim of this study was to understand the respective influence of dysfunctional parental interactions, SLEs, and attachment on the affective manifestations of BPD. First, we observed that the two types of adversity experiences (dysfunctional parenting and SLE) had distinct effects on affective symptoms, attachment, and emotional regulation. Although both types were correlated with affective symptoms: attachment and emotional regulation variables (hopelessness and alexithymia), these associations were not found in the same subscales and did not vary in the same direction. Second, BPD patients' emotional responses were quite different from the HCs' toward parent–child bonding and opposite to the HCs' for the response to SLE. Third, attachment dimensions analysis showed that they predicted the affective symptoms in HCs and BPD, but in BPD, their effect remained isolated, without any effect of parental interactions. In contrast, SLEs maintained their own influence, alongside attachment, on affective symptoms. Finally, the alexithymia subscales also revealed different associations with adversity in the HC and BPD groups, showing, in particular, an affective disconnection associated with SLE accumulation.

### Effect of Adversity on Affective Symptoms

We observed more parental dysfunction and SLEs in BPD than in controls, but the results of multivariate models also indicated an effect of maternal ALC behavior and SLEs on increased affective symptoms in the HC group. The role of childhood adversity events, including parental dysfunction and SLEs, is recognized in the pathogenesis of BPD: these events are dose-dependent risk factors in borderline features in affected children, adolescents, and adults, leading to more severe and an earlier onset of the disorder (7, 8, 41). Among these adversity factors, SLEs have been reported to be more prevalent in BPD than in other personality disorders or in major depressive disorder, leading to decreased psychosocial functioning over time (42). Early separations or losses are common in this population, which is characterized by high rates of breakups, chaotic family life, parental divorce, domestic violence, or a family history of psychiatric and substance abuse (43, 44). Family variables, such as parental hostility, inconsistency, or maternal overinvolvement, have also been described as specifically associated with an increased risk for early BPD symptoms (12, 45, 46). Our results are consistent with and extend further these studies, describing the effects of parental dysfunction and SLEs on the genesis of borderline symptomatology, and particularly on its affective symptoms, in a healthy population. The separate effects of low parental care (from either parent), higher control (maternal), and low level of affection (maternal) on affective symptoms reinforce previous observations, in which the effects of inconsistency and excessive parental involvement were both implicated in the development of the disorder (9, 47, 48).

### Discrepancies in HC and BPD Groups

In our study, the effects of adversity were different in the HC and BPD groups, and sometimes even opposite. Analyses in the BPD group showed no influence of dysfunctional parenting on affective symptoms, which may be surprising, as adversity in BPD is common and has been shown in the literature and, in our study, to have a strong influence on BPD symptoms in adolescents (49, 50). In our BPD group, SLEs also had a paradoxical negative effect on affective symptoms, reinforced by a parallel negative correlation with hopelessness. Beyond the logical loss of correlation between a risk factor in the general population and clinical variables in a pathological sample that may partly explain our results on parental dysfunction, paradoxical emotional manifestations in adult BPD subjects have been previously described (2, 5, 51, 52). They are understood as an adaptation to a specific socioemotional context including a threat in the environment (i.e., adversity factors in early relationships) (12). From their earliest historical clinical descriptions, BPD subjects are described as overreactive to minor events in their daily lives and often less responsive to major events (11, 53). BPD has also been associated with an impaired hypothalamic–pituitary–adrenal axis function. Some authors described continuous elevated cortisol production and blunted cortisol peaks following psychosocial stress in BPD patients (54). Dysregulation of the stress axis may reflect a mismatch between the environmental context and what the patient has been experiencing in his/her internal reality from an early age. The paradoxical lowering of affective symptoms in cases of cumulative SLEs can thus be understood as a protective desensitization in the face of an increasing number of traumas. This is consistent with studies showing a correlation between the severity of personality disorder and the severity of trauma exposure in adults (55, 56).

In our multivariate models, the effect of cumulative SLEs on affective symptoms appeared independent of attachment, suggesting a direct effect of adverse life experiences on affective regulation, for example, a direct effect on the axis of stress that causes affective symptoms. In this sense, evidence has emerged in the literature that there are direct psychopathological pathways between childhood abuse and borderline symptoms, not related to attachment, and involving or not emotional dysregulation (57). Fonagy and Luyten have suggested that the impact of SLEs on emotional dysregulation is a consequence of increased arousal and a lowered threshold for attachment and for deactivation of controlled mentalization (13). On the one hand, life events may well be part of a psychopathological pathway of their own. On the other hand, it turns out that adversity occurs within an already established attachment system that acts as a prism, where nuances of psychosocial interactions or SLEs are reduced to internal psychological perceptions derived from IWMs. The subject projects representations of self and others onto any situation, responsible for an “automatic” or anachronistic response, especially if these representations are rigid. IWMs are in essence based on repetition and do not change under occasional acute stress. Thus, we can assume that the meaning of the trauma is more or less consistent with the model of self and others, depending on the history of the borderline subject. And once the model of the self (and more or less the model of others) is altered, the occurrence of an adverse event is considered as something normal, that is, congruent. Thus, there is no longer a traumatic reaction in the sense of a psychological breakthrough or a strong emotional reaction. But then, the risk of recurrence increases in these patients, and it is often observed that relational warmth and positive life events are difficult to bear for adult borderline patients (58, 59).

### The Role of Attachment

The observation of distinct effects of adversity factors in BPD, and their different influences on emotional regulation in the HC and clinical groups seem to imply two levels of action of adversity and as such evoke the psychopathological model proposed by Fonagy and Luyten (13). These authors proposed to distinguish, in the genesis of BPD symptoms, between distal causes (early care, maltreatment) and proximal causes (stress sensitivity, attachment). These authors hypothesized a complex system including specific interactions at each level. Our results strongly support the idea that, initially or at a first level of intensity, the elements of adversity modify emotions directly but that in a second time and/or beyond a certain level of intensity, attachment, and the biological stress axis function on their own, relatively independently of external factors; in any case independently of the parent–child relationship. By showing in multivariate models that parental dysfunction correlates with affective symptoms in the control group and not with attachment but correlates with attachment in the BPD group and not with affective symptoms, our results suggest that, in BPD patients, affective symptoms are no longer rooted in dysfunctional relationships, but essentially in insecure attachment, which becomes a major determinant of emotional dysregulation.

The positive association we observed between SLEs and avoidant attachment in the bivariate correlations also questions the mechanisms underlying the many events experienced by borderline patients: early separations, parental physical or psychological illness, parental suicide attempts, disruptions and moves, placement, and so on. These experiences tend to be marked by separation and loneliness and refer to the links that have been established between unresponsive caregivers and the development of an avoidant attachment, a tendency to self-sufficiency to avoid rejection (15, 16). This is in line with our results in the HC group. But the negative bivariate correlations between SLEs and avoidant attachment that we observed in the BPD group suggest that, when a high level of anxiety characterizes attachment, the avoidance effect generated by SLEs might not apply. One hypothesis would be that the autonomy developed in the avoidant dimension of attachment requires relying on personal resources and a minimum positive self-model that BPD patients do not have, at least because of the parent–child dysfunction and perhaps for other reasons including genetic/personality aspects. Thus, the relationships established with caregivers would determine the effect of these one-time and repeated life events, not only on stress but also on attachment itself. Multivariate regressions performed on the anxiety and avoidance dimensions of attachment support this hypothesis, by showing that the effect of adversity on attachment is marked primarily by the role of the parents and not by SLE. Individuals with BPD thus vacillate between an intense need for closeness and failed attempts at self-sufficiency (17, 18, 23), and SLEs may reinforce this oscillation through an increased need for self-sufficiency that returns the borderline patient to his/her strong dependence. When in this deadlocked system new life events occur, BPD patients seem to deactivate their emotional and relational distress to some level, with, as a last resort, numbing and disconnection from any form of emotion.

### Alexithymia and Adversity

Alexithymia measures were associated with adversity in HCs and BPD, but there again, differently among groups. In the HC group, lower levels of parental care (from either parent) were correlated with DIF. This result echoes literature data in the general population and is in line with clinical observations, proposing that childhood trauma may have an impact on the development of alexithymia in young adults (60). Since the first clinical observations of alexithymia in psychosomatic disorders, several models have been proposed about its origin, including hypotheses of a possible role of traumatic experiences and/or a dysfunctional parent–child relationship (25, 61). Of the four parental bonding factors examined, the strongest and most consistent pattern of correlations involved maternal factors, and a meta-analysis reported strong evidence of maternal care influence on DIF and to a lesser extent on DDF in adults (62, 63). Moreover, additional data from adult clinical samples highlighted a negative influence of maternal and paternal care on DIF and DDF (64, 65). Significant positive associations have also been identified between maternal and paternal overprotection and alexithymia in clinical diagnoses (66). Together, these findings suggest a differential impact of maternal and paternal bonding on alexithymia, DIF, and DDF across general and clinical samples. It was assumed that the role of the father was all the more fundamental in pathological situations where the link to the mother was dysfunctional. It was supposed to be more important in relation to dysfunctional coping and negative affect (63). Finally, it has been highlighted that an optimal parenting style in one parent buffered the effect on alexithymia in a clinical sample, suggesting that further research is needed to explore the combined effects of maternal and paternal parenting styles on alexithymia (66). Our results, by showing that a low level of paternal care may increase DDF only in the BPD group, suggest that father responsiveness is even more central in the borderline group when the mother is not receptive to the emotions expressed by the child. This hypothesis is consistent with the observations suggesting that a BPD diagnosis in adolescents was best predicted by a combined disorganization toward each parent, namely, insecurity toward the father and deactivation of the attachment system in the relationship with the mother (67). It also echoes our observation of an exclusive correlation between maternal ALC and avoidant attachment, as well as paternal ALC and anxious attachment (models 5 and 6) in the BPD group.

The EOT subscale was not correlated with affective symptoms or to hopelessness. Cautious interpretation about EOT is often suggested in studies on alexithymia, because of the relatively weak psychometric properties of the EOT subscale (68). But in our study, the paradoxical positive correlation between this subscale and SLEs may be of interest, as we observed that SLEs modify emotional regulation in two steps. In the BPD group, where cumulative SLEs showed an affective disconnection effect, their correlation with the EOT subscale makes sense. Indeed, the EOT describes the process by which a subject avoids his/her emotional functioning by focusing attention on the operative, concrete, external world. At a high level of cumulative adversity and emotional dysregulation, a high alexithymia score would therefore represent the loss of all forms of emotionality and suffering. The negative correlation between SLEs and hopelessness scores suggests that this operant functioning is a defense mechanism put in place to protect oneself from repetitive trauma in a survival logic developed by BPD adolescents.

Some limitations must be considered when evaluating the results of this study. Individuals with alexithymia lack self-reflective capability and emotional awareness (69). Therefore, the validity of measuring alexithymia, parent–child bonding, and attachment through their subjective assessment may be questioned. Although the use of the PBI, RSQ, and TAS scales is common in these populations, future research should focus on the use of both objective and subjective measures to assess these variables in order to detect potential discrepancies (70). Second, our distinction between parent–child bonding and SLEs is schematic and has limitations in that these life events include many situations that reflect or influence the quality of the parent–child bond. BPD factors tend to cumulate, and their differentiation necessarily presents a somewhat artificial reduction. Moreover, among these adversity factors, maltreatment is not considered in this study, even though it represents an important factor in the history of BPD patients (41). As our study was centered on emotional regulation, we wanted to focus on adversity factors that were homogeneous in their modality (parent–child relationships were constant, and SLE, in their repetition, had factors that were comparable between them). Conversely, maltreatment includes durable and stable factors (such as physical neglect or emotional abuse) or punctual factors (such as sexual abuse), which makes a study on emotional regulation even more complex. These factors would therefore merit their own analysis, given their internal heterogeneity. Moreover, the missing data in the BPD group have impacted the choice of statistical models. They are probably related to the legal status of this research, which allowed the financial compensation of HCs but not of patients, for their participation in the study. This difference had a visible effect on the motivation of patients and controls to fill in the questionnaires, which were lengthy. We did not compare the severity of BPD patients who did or did not complete the questionnaires, and this is another limitation to our study. Finally, analyses including BPD symptoms were limited to affective characteristics and did not assess impulsivity and relational and cognitive dimensions, which also may be strongly correlated with attachment. Further studies would also be needed to clarify whether the emotional disconnection we observed in BPD patients with cumulative adversity corresponds to a reduction in biological physical stress, reported physical stress, or only an emotional disconnection with intact physiological conditions, as our study did not distinguish between these elements.

Despite these limitations, our results confirm that the alteration of the attachment system in BPD participates to the dysregulation of emotions, independently of the environmental context. It appears that dysfunctional parent–child relationships do not induce additional affective symptoms and hopelessness once attachment is dysregulated. It also appears that the accumulation of SLEs even decreases this sense of hopelessness, alongside the development of an emotional numbing response with operant functioning. This may reflect coping strategies to protect the extreme fragility of the sense of self in these patients.

## Data Availability Statement

The datasets presented in this study can be found in online repositories. The names of the repository/repositories and accession number(s) can be found below: https://doi.org/10.6084/m9.figshare.14885235.v1.

## Ethics Statement

The studies involving human participants were reviewed and approved by the reference Ethics Committee in Paris, Cochin hospital, approved the study (Authorization No. 0611259). Written informed consent to participate in this study was provided by the participants' legal guardian/next of kin.

## Author Contributions

Conceptualization was performed by MC, MS, and AP-S. Methodology was performed by MS and AP-S. Material preparation, investigation, and data collection were performed by MR, JB, VP, and JS. Formal analysis was performed by JS, GS, and MR. Original draft preparation was performed by MR and JB, and reviewed by MS, AP-S, VP, and MC. The resources were found by MC and AP-S. Supervision was performed by MC. The manuscript was written by MR and JB and approved by all authors. All authors contributed to the study conception and design.

## Funding

This research was supported by a grant from the Wyeth Foundation for Child and Adolescent Health and by a grant from the Eli Lilly Foundation. The funders had no role in study design, data collection and analysis, decision to publish, or preparation of the manuscript.

## Conflict of Interest

The authors declare that the research was conducted in the absence of any commercial or financial relationships that could be construed as a potential conflict of interest.

## Publisher's Note

All claims expressed in this article are solely those of the authors and do not necessarily represent those of their affiliated organizations, or those of the publisher, the editors and the reviewers. Any product that may be evaluated in this article, or claim that may be made by its manufacturer, is not guaranteed or endorsed by the publisher.
