# Corrigendum: Paradoxes in borderline emotional dysregulation in adolescence: influence of parenting, stressful life events, and attachment

**DOI:** 10.3389/fpsyt.2024.1408042

**Published:** 2024-05-22

**Authors:** Marion Robin, Jean Belbèze, Alexandra Pham-Scottez, Gérard Shadili, Victoire Peres, Jérôme Silva, Maurice Corcos, Mario Speranza

**Affiliations:** ^1^ Department of Adolescent and Young Adult Psychiatry, Institut Mutualiste Montsouris, Paris, France; ^2^ Medical School, Paris Descartes University, Medical School, Paris, France; ^3^ University Hospital Group, Paris Psychiatry and Neurosciences, Paris, France; ^4^ Versailles General Hospital, Le Chesnay, France; ^5^ Paris-Saclay University, UVSQ, CESP, INSERM, Gif sur Yvette, France

**Keywords:** borderline, adolescent, attachment, alexithymia, parental bonding (PBI), stressful life events

In the published article, there was an error in the legend for **Figure 1** as published.

**Figure 1:**
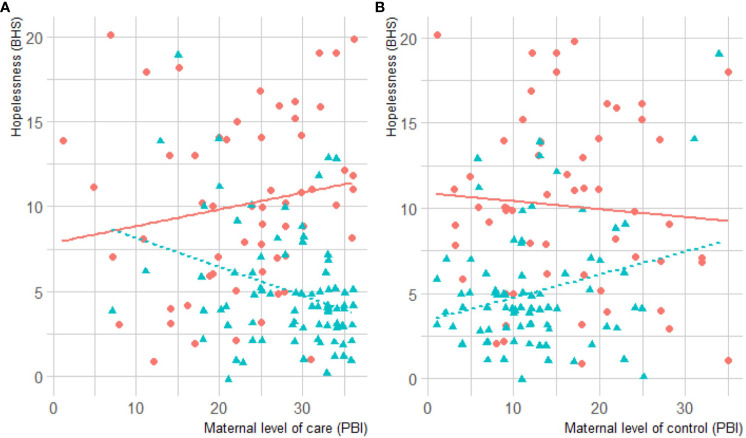
**(A)** Correlation between maternal care and hopelessness. **(B)** Correlation between maternal control and hopelessness in BPD and HC groups. BPD scores are represented in red, and HC’s in blue. BHS, Beck Hopelessness Scale; BPD, Borderline Personality Disorder; HC, healthy controls.

The sentence “BPD scores are represented in blue, and HC’s in red” is incorrect.

The corrected legend appears below.

“BPD scores are represented in red, and HC’s in blue”

The authors apologize for this error and state that this does not change the scientific conclusions of the article in any way. The original article has been updated.

